# Inconsistency between serum IgG κ and glomerular IgA λ chain types in proliferative glomerulonephritis with monoclonal deposits successfully treated with daratumumab-based therapy

**DOI:** 10.1097/MD.0000000000044607

**Published:** 2026-02-13

**Authors:** Huihui Chen, Duqun Chen, Guolei Zhang, Meijuan Cheng, Jingjing Jin, Zhezhe Niu, Rongfang Zhu, Liping Guo, Jiawei Wang, Yaling Bai, Jinsheng Xu, Zhen Cheng

**Affiliations:** a Department of Nephrology, Hebei Clinical Research Center for Chronic Kidney Disease, Hebei Key Laboratory of Vascular Calcification in Kidney Disease, The Fourth Hospital of Hebei Medical University, Shijiazhuang, Hebei, China; b National Clinical Research Center for Kidney Diseases, Jinling Hospital, Affiliated Hospital of Medical School, Nanjing University, Nanjing, Jiangsu Province, China; c Department of Orthopedics, The Third Hospital of Hebei Medical University, Shijiazhuang, Hebei, China.

**Keywords:** case report, daratumumab, monoclonal gammopathy of renal significance, proliferative glomerulonephritis with monoclonal immunoglobulin deposits

## Abstract

**Rationale:**

Here we report a rare case of a patient with serum immunoglobulin G (IgG) κ and glomerular immunoglobulin A (IgA) λ chain types in proliferative glomerulonephritis with monoclonal immunoglobulin deposits (PGNMID). The patient showed clinical improvement after treatment with daratumumab combined with bortezomib and dexamethasone. To date, data on daratumumab use in patients with PGNMID are limited, and its treatment remains challenging.

**Patient concerns:**

We report a rare case of a 50-year-old male patient with PGNMID in whom serum kappa free light chain (FLC) and lambda were significantly higher than average, and immunoglobulins were IgG-kappa and FLC-kappa types. The Bence Jones protein test was positive, and the urine immunofixation electrophoresis revealed a kappa light-chain type.

**Diagnoses:**

Renal biopsy was performed after admission. The pathological diagnosis was IgA λ-type PGNMID combined with acute tubulointerstitial injury and chronic disease.

**Interventions:**

The patient became seriously ill upon admission and progressed rapidly. After administration of the first dose of daratumumab at 100 mg, 3 courses of bortezomib (1.3 mg/m^2^) were administered in combination with dexamethasone (10 mg) for 21 days.

**Outcomes:**

After 3 sessions of treatment, the patient’s blood creatinine level was stable at 300 μmol/L, urine volume was normal, and serum kappa FLC was reduced to 477 mg/L.

**Lessons:**

This rare case of PGNMID showed a discrepancy between serum IgG κ and glomerular IgA λ chain. Treatment with daratumumab, bortezomib, and dexamethasone significantly reduced serum FLC levels and stabilized the blood creatinine level. Daratumumab is a promising drug for the treatment of PGNMID.

## 1. Introduction

Proliferative glomerulonephritis (GN) with monoclonal immunoglobulin deposits (PGNMID) is a recently described entity in the spectrum of monoclonal gammopathy of renal significance (MGRS).^[1–4]^ PGNMID was first described by Nasr et al (2004). This disease is uncommon, with an autologous renal biopsy rate of 0.17 to 0.21%, as reported by Nasr et al.^[5,6]^ It is characterized by hyperplastic lesions observed under a light microscope. Only single-subtype immunoglobulin G (IgG) and single light-chain deposits were observed using immunofluorescence staining, and granular electron-dense matter was observed under an electron microscope. The detection rate of nephrotoxic monoclonal immunoglobulin and abnormal bone marrow B-cell clones in the serum or urine of patients with PGNMID is only 30%. Moreover, the renal prognosis is poor, with 25% of these patients progressing to end-stage renal disease within 30 months.^[7,8]^ PGNMID pathophysiology remains unclear, and its treatment is challenging. In 2019, Frank et al^[9]^ reported that PGNMID variants with monotypic LC, immunoglobulin A (IgA), or IgM depositions are uncommon. With the deepening of our understanding of PGNMID and the increasing number of cases, discrepancies in serum and glomerular deposits have been observed.^[10–13]^ Despite the limited evidence, the current consensus is that PGNMID treatment should target potential clones. However, heterogeneity in PGNMID prognosis is plausible, and current therapies targeting potential clones remain controversial.^[14–17]^ We herein report a case of PGNMID with inconsistency between serum IgG κ and glomerular IgA λ chain-type deposits that were successfully treated with daratumumab combined with bortezomib and dexamethasone.

## 2. Case presentation

**Figure 1.**
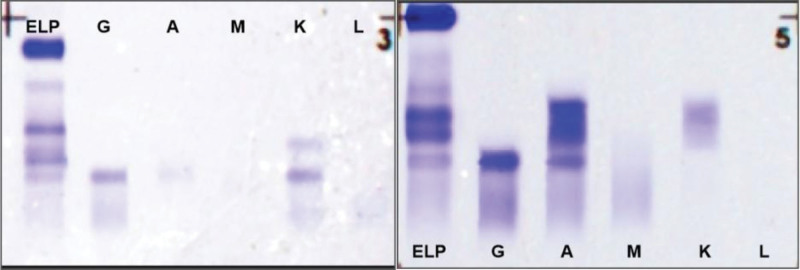
Results of hematuria immunofixed electrophoresis. The left picture shows the results of hemoimmunofixed electrophoresis. On the right is the result of urine immunofixation electrophoresis.

We report the case of a 50-year-old male patient with a history of urinary protein deposits for >1 year and elevated serum creatinine levels over the last 4 months. At presentation, urinalysis showed urine red blood cell count and protein level of 1162.4 red blood cells/μL and 15.29 g/day, respectively. Serum creatinine, serum albumin, globulin, β2-microglobulin, calcium, and estimated glomerular filtration rate were 434.9 μmol/L, 28.9 g/L, 15.9 g/L, 14.2 mg/L (significantly increased), 1.94 mg/dL, 13 mL/min/1.73 m^2^, respectively. Serum free light chain (FLC) detection results showed a kappa FLC, lambda FLC, and κ/λ of 4900 mg/L, 27.2 mg/L, and 180.15, respectively; κ/λ was significantly higher than average. During hospitalization, 2 bone marrow punctures showed mature plasma cells at concentrations of 0.5% and 7% (Tables S1 and S2, Supplemental Digital Content, https://links.lww.com/MD/R318). In addition, the patient underwent external examination for multiple myeloma immunotyping, chronic lymphocytic leukemia and lymphoma immunotyping. The results showed that a total of 245,975 nucleated cells were collected and analyzed, among which 4886 were monoclonal plasma cells, accounting for 2.0% of the total nucleated cells, and when analyzing 8.5% of the mature lymphocyte population, no abnormal expression was observed. Only a suspected plasma cell population of 1.9% was identified (Figs. S1 and S2, Supplemental Digital Content, https://links.lww.com/MD/R318). Serum immunofixation electrophoresis showed precipitation bands in the IgG lane, with 2 precipitated bands in the kappa lane; the immunoglobulins were IgG-kappa and FLC-kappa types. The patient was urine Bence Jones protein (BJP)-positive, and urine immunofixation electrophoresis suggested a kappa light chain type (Fig. 1).

The patient was 167 cm tall, weighed 63 kg, and had a body mass index of 22.59. His blood pressure was 171/101 mm Hg. The patient had a history of hypertension in the last 3 to 4 years (up to 190–200/100–110 mm Hg), with poor blood pressure control. Although physical examination did not reveal any obviously enlarged lymph nodes or hepatosplenomegaly, head computed tomography revealed bilateral maxillary and frontal sinusitis. Multiple cysts were found in the liver, with slight pleural effusion. A chest computed tomography scan revealed scattered inflammation in the right and left inferior lung lobes. A few fibrous cords in the lower lobe of the right lung and aortic wall calcifications were observed. Bone biopsy showed active bone marrow hyperplasia with minimal monoclonal plasma cell infiltration. The laboratory data of the patient are presented in Table 1. His urinary protein level was 15.29 g/day, with massive microscopic hematuria, and urine immunofixation suggested BJP-κ type. Serum immunofixation showed IgG-κ type, and the blood immunoglobulin FLC κ/λ ratio was also elevated (Table 1).

**Table 1:** Laboratory data on admission.

Urinary examination		Blood chemistry	
PH (4.5–7.5)	6	HbA1c (%, <6.2)	6
Protein (g/d)	15.29	Glu (mmol/L, 4.1–5.9)	5.4
Occult blood (/µL)	1162.4	ALB (g/L, 35–50)	23
Glucose	3+	BUN (mmol/L, 3.2–7.1)	14
NAG (U/L, 0–11.5)	23.7	SCr (μmol/L, 57–111)	434.9
**Complete blood count**		eGFR (mL/min/1.73 m^2^)	13
WBC (×10^9^/L, 3.5–9.5)	5.61	UA (mg/dL, 3.7–7.8)	396
RBC (×10^12^/L, 4.3–5.8)	3.15	Na (mEq/L, 138–145)	137
Hgb (g/L, 130–175)	94	K (mEq/L, 3.6–4.8)	4.4
Hct (%, 40–50%)	28.4	Cl (mEq/L, 101–108)	109
PLT (×10^9^/L, 125–350)	292	Ca (mg/dL, 8.8–10.1)	2.01
**Serology**		AST (U/L, 13–30)	19
ANA	<1:100	ALT (U/L, 10–42)	26
MPO-ANCA (AU/mL, <20)	<20	LDH (U/L, 124–222)	204
Anti-ds-DNA (U/mL, <1:10)	<1:10	ALP (U/L, 106–322)	59
C3 (g/L, 0.7–1.4)	0.566	γGTP (U/L, 0–50)	14
C4 (g/L, 0.1–0.4)	0.142	T-Bil (µmol/L, 3–22)	5.6
ASO (IU/mL, <116)	<25	T-Chol (mmol/L, 3–6)	6.21
RF (IU/mL, <20)	<20	TG (mmol/L, 0.28–2.2)	3.34
FLC κ (mg/L, 3.3–19.4)	4900	CRP (mg/L, 0–8)	0.5
FLC λ (mg/L, 5.71–26.3)	27.2	IgG (g/L, 7.51–15.6)	3.3
FLC κ/λ (0.37–3.1)	180.15	IgA (g/L, 0.82–4.53)	0.332
uIFE	BJP-κ	IgM (g/L, 0.46–3.04)	<0.0417
sIFE	IgG-κ	IgE (IU/mL, <190)	38.1
PLA2R (RU/mL, <20)	<2	β2mg (mg/L, 1–2.3)	14.2

ALB = albumin, ALP = alkalinephoshoatase, ALT = alanine aminotransferase, ANA = antinuclear antibody, ASO = antistreptolysin O, AST = aspartate aminotransferase, BUN = blood urea nitrogen, Ca = calcium, CRP = C-reactive protein, eGFR = estimated glomerular filtration rate, FLC = free light chain, Hct = hematocrit, Hgb = hemoglobin, K = potassium, LDH = lactate dehydrogenase, MPO-ANCA = myeloperoxidase anti-neutrophil cytoplasmic antibody, Na = sodium, NAG = N-acetyl-β-D-glucosaminidase, PLA2R = phospholipase A2 receptor, PLT = platelets, RBC = red blood cells, RF = rheumatoid factor, SCr = serum creatinine, SG = specific gravity, sIFE = serum immunofixation electrophoresis, TG = triglyceride, UA = uric acid, uIFE = urine immunofixation electrophoresis, WBC = white blood cells, β2mg = β2-microglobulin.

**Figure 2.**
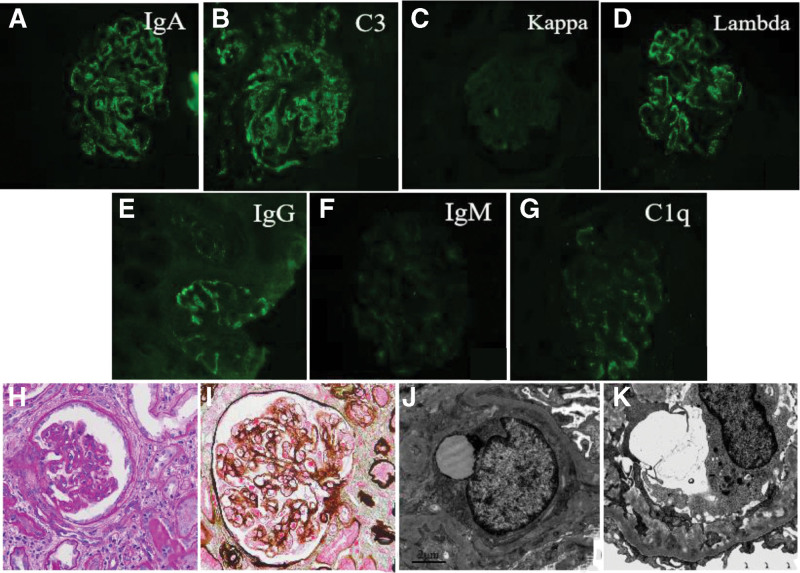
Renal biopsy findings. (A–G) Immunofluorescence of renal biopsy showed IgA (3+), C3 (3+), k light chain trace, λ light chain (3+) diffuse deposition in granular form in the mesangial region and vascular loops (×200). (H and I) Light microscopy of renal puncture tissue revealed occasional rubiophil in the subcutaneous and epithelial side of the glomerular capillary loops, and more peripheral loops were stratified. (×400). (J and K) Electron microscopic photograph of the renal biopsy, showing the electron-dense deposits in mesangial areas (×6000).

**Figure 3.**
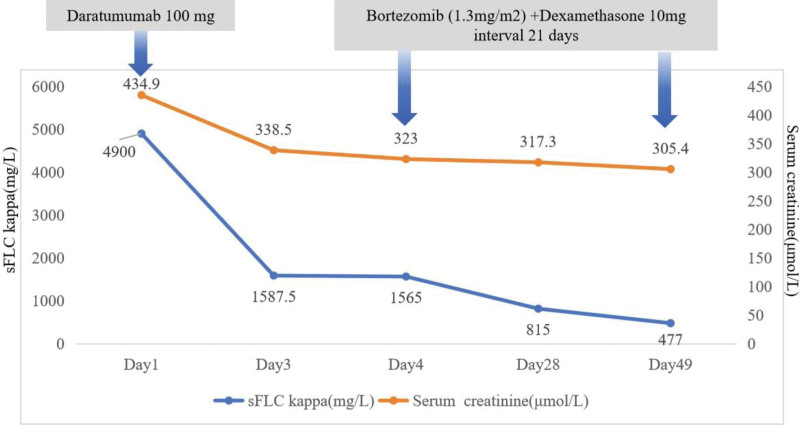
The patient’s outcome of treatment after diagnosis. sFLC kappa = serum-free light chain kappa.

Pathological examination of the renal biopsy (Fig. 2) showed 32 glomeruli, 15 glomerular abandons, 6 segmental scleroses, and false tubule formation. The mesangial area of the residual glomeruli was slightly widened. The mesangial cells and matrix increased, the capillary loops were not well opened, the peripheral segment loops were attached to the capsule wall, the capsule wall segments were thickened and stratified, and pericystic fibrosis was observed. PASM-Masson staining suggested occasional rubiophils on the subcutaneous and epithelial sides of the glomerular capillary loops, and more peripheral loops were stratified. Severe chronic renal tubulointerstitial disease was observed, with multifocal tubular vacuolar degeneration, scattered cholesterol crystals, interstitial diffuse mononuclear cells, a small number of plasma cells, neutrophil infiltration, little foam cell distribution, and interstitial fibrosis ++ status. Arteriolar segmental hyaline degeneration was observed, and the interlobular arterial elastic layer was thickened and stratified. Types and characteristics: glomerular membrane hyperplasia with IgA deposition, globular waste (46.88%), segmental sclerosis (18.75%), severe chronic tubulointerstitial disease (55%), mild acute disease (15%), hyaline degeneration, and arteriosclerosis. Congo red staining of the renal tissue was not positive. Immunofluorescence of the renal biopsy showed IgA (3+), C3 (3+), k light chain (trace), λ light chain (3+), and diffuse deposition in granular form in the mesangial region and vascular loops. Trace IgM and negative IgG were observed. Fibrin was negative. Electron microscopy (Fig. 2) revealed a widened mesangial region, increased mesangial cells and matrix, and dense matter with high electron density deposited in the mesangial region. The glomerular capillary loops were well opened, and inflammatory cells were clustered in the loops. Insertions and new basal membranes were formed in many loops, and the subcutaneous area within the segmental basal membrane was widened and loosened. Dense matter with high electron density was also distributed under the subcutaneous membrane and inserted into the basal membrane. Moreover, no electron-dense matter was deposited on the epithelial side. Glomerular podocytes showed 60 to 70% foot process fusion, a small amount of microvillous cytoplasm, intracytoplasmic vacuoles, and lysosomal phagocytosis. The brush edges of the proximal renal tubule epithelium were sparse and exfoliated, with intracytoplasmic vacuoles, lysosomal particles, and high inflammatory cell infiltration in the interstitium. The Congo red staining result was negative. Based on the above findings, a diagnosis of IgA λ-type PGNMID and acute tubulointerstitial injury combined with chronic disease was made. After administering the first dose of daratumumab (100 mg), the serum kappa FLC levels decreased from 4900 mg/L to 1587.5 mg/L. However, the patient developed a fever reaction 1 hour after the infusion of daratumumab, with a body temperature of 38°C, no other accompanying symptoms, and low inflammatory indicators. Considering an allergic reaction to the drug, 3 courses of bortezomib (1.3 mg/m^2^) combined with dexamethasone (10 mg) were administered for 21 days. At present, the patient’s blood creatinine was basically stable at 300 μmol/L; urine volume was normal, and serum kappa FLC was reduced to 477 mg/L (Table 2, Fig. 3). The recombinant cluster of differentiation38 monoclonal antibody daratumumab combined with bortezomib and dexamethasone may be a new and effective drug option for patients with PGNMID

**Table 2:** The laboratory indicators of the patient before and after treatment.

Date	SCr (μmol/L)	Proteinuria (g/d)	sFLC κ (mg/L, 3.3–19.4)	sFLC κ/λ (0.37–3.1)
2023/5/6	434.9	15.29	4900	180.15
2023/5/9	338.5	/	1587.5	113.39
2023/5/10	323.0	/	1565	106.25
2023/5/24	316.9	11.16	1245	87.7
2023/6/5	317.3	8.56	815	60.5
2023/6/26	305.4	3.48	477	18.4

SCr = serum creatinine, sFLC κ/λ = serum-free light chain kappa/lambda, sFLC κ = serum-free light chain kappa.

## 3. Discussion

Herein, we report a case of PGNMID and describe differences in the heavy and light chains between the serum and kidney. Although this case showed IgG-κ and BJP-κ in serum and urine, bone marrow examination suggested a diagnosis of multiple myeloma. Renal biopsy under light microscopy revealed glomerular membrane proliferative lesions. Immunofluorescence studies revealed particle deposition of IgA, C3, and λ light chains in the mesangial region and capillary walls. Congo red staining was negative, and electron microscopy revealed dense deposits with high electron density in the mesangial region, subcutaneous area, and basement membranes. No electron-dense deposits were observed on the epithelial side. The brush edges of the proximal renal tubule epithelial cells were sparse and exfoliated, and high levels of inflammatory cell infiltration in the interstitium were observed. Based on these results, the patient was diagnosed with glomerular membrane hyperplasia, acute tubulointerstitial injury, and a chronic disease.

The term MGRS was introduced by the International Kidney and Monoclonal Gammopathy Research Group (IKMG) in 2012. The IKMG updated the definition of MGRS in 2017. Within MGRS, PGNMID is a particular category. The disease was first identified in 2004 and has a short research history. Compared to other MGRS types, the detection rate of circulating clones in patients with PGNMID is low, and it is easy to miss and misdiagnose clinically. PGNMID is associated not only with MGRS but also with malignant tumors, infections, and other factors. In clinical settings, 65 to 100% of PGNMID cases are associated with MGRS.^[12]^ In a recent article by Shoko Miura,^[10]^ a PubMed database search for “glomerulonephritis with monoclonal IgG deposition” or “hyperplastic glomerulonephritis with monoclonal immunoglobulin deposition” found 81 articles. These articles showed differences in heavy and/or light chains in serum and kidneys in 8 out of 204 cases. One patient was reported to have IgA-k deposition in the glomeruli and IgG-k deposition on peripheral serum immunofixation electrophoresis. However, this is the first case report of IgA-λ deposition in the glomeruli of a patient with PGNMID and IgG-k deposition suggested by peripheral serum immunofixation electrophoresis. Although the significance of the discrepancy in κ and λ is not clear, IgG3 is characterized by a strong positive charge and large molecular weight, which might explain its affinity for glomeruli.^[18]^ At present, the pathogenesis of PGNMID remains unclear. According to the mechanism of immunoglobulin production and the known clinical manifestations of PGNMID, Manna Li et al^[3]^ proposed the following hypothesis about its etiology: Abnormal immunoglobulins are secreted by malignant B cells or plasma cells in the bone marrow or serum; Normal B or plasma cells in the bone marrow or serum are affected by various factors, resulting in abnormal immunoglobulin secretion. The first condition is closely related to kidney damage caused by hematological malignancies.

After reviewing the literature, PGNMID with only monoclonal immunoglobulin LC deposition only (LC-PGNMID) was found to be associated only with malignancy. The prognosis is generally poor, and more than 50% of these patients show gradual progression to chronic renal insufficiency or end-stage kidney disease. In studies by Nasr et al,^[5,6]^ some patients with MGRS-related PGNMID had stable renal function at the last follow-up after treatment with renin-angiotensin system inhibitors or prednisone alone, even when they had nephrotic-range proteinuria or were at stages 3 and 4 of chronic kidney disease, confirmed by renal biopsy. As there is no definitive treatment for PGNMID, renin-angiotensin system inhibitors are considered reasonable treatments for this type of disease. Initially, patients treated with valsartan alone experienced a decrease in urinary protein levels; however, proteinuria gradually increased, and renal insufficiency was noted. Recently, Gumber et al^[8]^ reported that the principle of treating MGRS-related PGNMID is by targeting potential clones. Studies have shown that patients with MGRS-associated PGNMID with advanced chronic kidney disease can achieve stable disease with conservative treatment or by targeting potential clones.^[19,20]^ Therefore, the need to target potential clones in patients with MGRS-associated PGNMID remains inconclusive. Some researchers have noted that PGNMID clones are most often related to plasma cell clones (60–100%). Some cases of bortezomib-based therapy have been reported, with most patients showing improvement.^[21,22]^ However, bortezomib and dexamethasone treatment alone may not be effective in some cases. Therefore, it is unclear whether the current novel CD38 monoclonal antibody, daratumumab, is effective against PGNMID. After a literature review of relevant articles published in the last 20 years, few cases have been treated with the novel CD38 monoclonal antibody against PGNMID.

In the above case, the serum kappa FLC decreased from 4900 mg/L to 1587.5 mg/L after the administration of daratumumab at 100 mg. However, the patient developed a fever reaction 1 hour after the infusion of daratumumab, with a body temperature of 38°C, no other accompanying symptoms, and low levels of inflammatory indicators. Considering the allergic reaction to the drug, 3 courses of bortezomib (1.3 mg/m^2^, d 1, 4, 8, 11) combined with dexamethasone were administered. The patient has undergone follow-up for 3 months, and his blood creatinine has stabilized at 300 μmol/L, urine volume was normal, and serum kappa FLC has decreased to 477 mg/L. We predict that the new CD38 monoclonal antibody, daratumumab, may be an effective drug option for patients with PGNMID.

## Author contributions

**Data curation:** Duqun Chen, Jiawei Wang.

**Formal analysis:** Duqun Chen, Guolei Zhang, Meijuan Cheng, Jingjing Jin, Zhezhe Niu, Rongfang Zhu, Yaling Bai, Jinsheng Xu.

**Methodology:** Duqun Chen, Guolei Zhang, Meijuan Cheng, Liping Guo, Yaling Bai, Jinsheng Xu.

**Project administration:** Zhen Cheng.

**Writing – original draft:** Huihui Chen.

**Writing – review & editing:** Duqun Chen, Zhen Cheng.

## Supplementary Material


