# Supplementary material for: Inconsistency between serum IgG κ and glomerular IgA λ chain types in proliferative glomerulonephritis with monoclonal deposits successfully treated with daratumumab-based therapy
Source: Medicine (Baltimore). 2026 Feb 13;105(7):e44607. doi: 10.1097/MD.0000000000044607 (PMC12908815; doi:10.1097/MD.0000000000044607)
Supplement: Supplementary file 1 [file medi-105-e44607-s001.pdf]

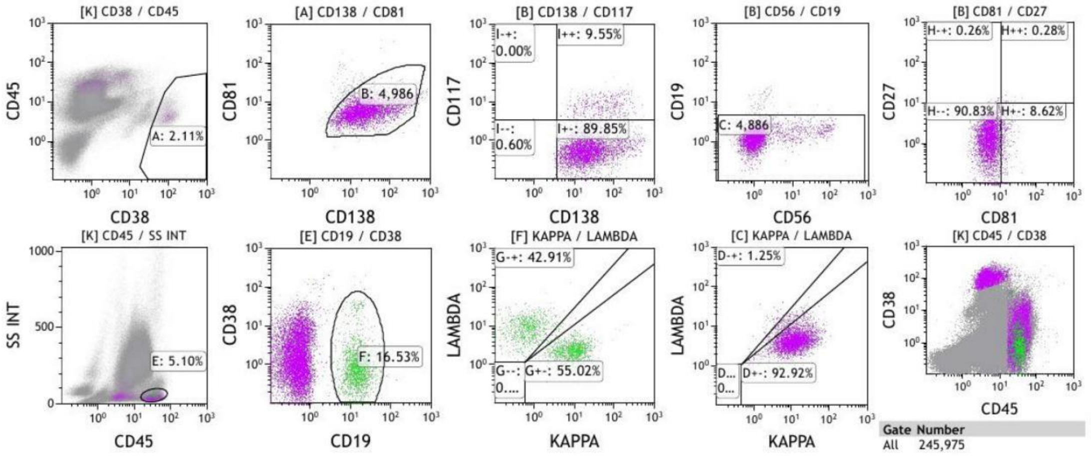

Supplementary Figure 1. The patient's report of Multiple Myeloma Immunophenotyping. The above results showed that cKAPPA, CD138, and CD38 are expressed, while CD45 was weakly expressed. cLAMBDA, CD19, CD56, CD117, CD27 and CD81 were not expressed. A total of 245,975 nucleated cells were collected and analyzed, among which 4,886 were monoclonal plasma cells, accounting for 2.0% of the total nucleated cells.

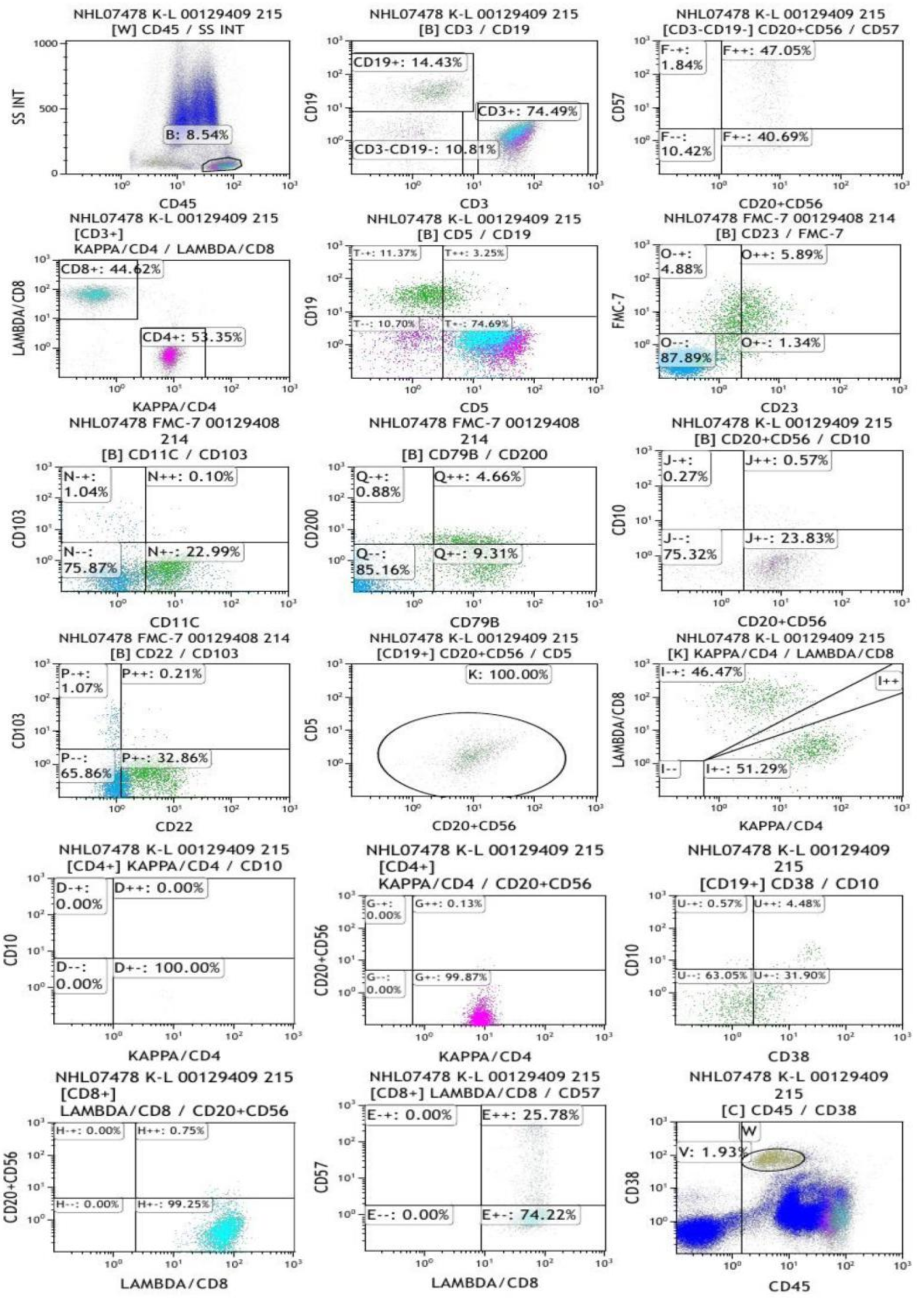

Supplementary Figure 2. The patient's report on Immunophenotyping of Chronic Lymphocytic Leukemia and Lymphoma by Flow Cytometry. The above results showed that there were no abnormalities in the phenotypes of B lymphocytes, T lymphocytes, and NK cells. When analyzing 8.5% of the mature lymphocyte population, no abnormal expression was observed. Additionally, a suspected plasma cell population of 1.9% was identified.
